# Urinoma: A Rare but Potential Differential of Acute Abdomen

**DOI:** 10.7759/cureus.67368

**Published:** 2024-08-21

**Authors:** Tooba Anjum, Maryam Ikram, Rehma Abdulhameed, Asad Khan, Zeeshan R Mirza

**Affiliations:** 1 Diagnostic Radiology, Institute of Nuclear Medicine and Oncology Lahore (INMOL) Cancer Hospital, Lahore, PAK; 2 Nuclear Medicine and Allied Sciences, Swat Institute of Nuclear Medicine Oncology and Radiotherapy (SINOR) Cancer Hospital, Swat, PAK

**Keywords:** mri, ureteral trauma, spontaneous ureteral injury, urinoma, case report

## Abstract

Urinoma is an encapsulated collection of extravasated urine in the retroperitoneal space. This rare and potentially dangerous event can occur spontaneously or iatrogenically varying from asymptomatic cases to acute abdomen. The initial diagnostic modality is ultrasound. Suspicious ultrasound findings are confirmed by CT or MRI. Treatment depends on the inciting factor and varies from conservative management to interventional drainage procedures.

We report the case of a male patient with known urothelial carcinoma of the urinary bladder who presented acutely with a complaint of progressive painful swelling in the right lumbar region. Imaging revealed a breach in the right ureter leading to urinary extravasation and the formation of progressively enlarging urinoma extending to the contralateral lumbar region. Based on radiological diagnosis, the patient was urgently referred to a urologist.

Spontaneous renal rupture should be one of the differentials of a patient presenting with acute abdomen as it can lead to a rare and potentially threatening condition of urinoma.

## Introduction

Encapsulated collection of extravasated urine in peri-renal or para-ureteral space is referred to as urinoma which is a rare pathological event. It mostly occurs secondary to obstructive uropathy, especially in newborns, trauma, pelvic tumor, or iatrogenic, as seen in gynecological and urological procedures [1,2]. The condition can also be rarely seen in retroperitoneal fibrosis, pregnancy, or abdominal aortic aneurysm. Renal rupture can be parenchymal or of the renal collecting system. The resultant retroperitoneal urinoma is a potentially dangerous condition. Patients can be clinically occult or may present with acute abdomen. Diagnosis is based on imaging. Ultrasonography raises the suspicion of urinoma based on gray-scale findings that are confirmed by CT or MRI [3]. Here, we report the case of a 50-year-old man with iatrogenic right ureteral breach and resultant peri-ureteral urinoma that extended to the contralateral lumbar region as well.

## Case presentation

A 50-year-old male patient, a known case of urinary bladder carcinoma, presented to our oncology outpatient department with a complaint of hematuria and acute-onset diffuse pain and swelling in the right abdominopelvic region for two days after a second transurethral resection of bladder tumor (TURBT). The swelling had progressed over time. The patient was vitally stable and afebrile. There was no history of trauma or similar attacks of painful swelling. The patient’s cystoscopy eight months ago revealed three soft tissue urinary bladder masses with no invasion of the muscle layer (T1 N0 M0). He underwent transurethral resection of bladder cancer with excision of all these masses. The second TURBT was performed 10 weeks after the initial TURBT by the same surgeon after the patient developed hematuria and right flank pain. Imaging revealed tumor recurrence near the right ureterovesical junction with subsequent development of right hydroureteronephrosis. The resection included visible residual/recurrent tumor. The patient was then given intravesical immunotherapy and followed up by cystoscopy and upper urinary tract ultrasonography. The patient had a history of multiple TURBT and was on chemotherapy.

On physical examination, a swelling was observed in the right lumbar region that was firm and tender on palpation with undefined boundaries. Bowel sounds were normal. Laboratory evaluation showed normal hemoglobin and white cell count.

**Figure 1:**
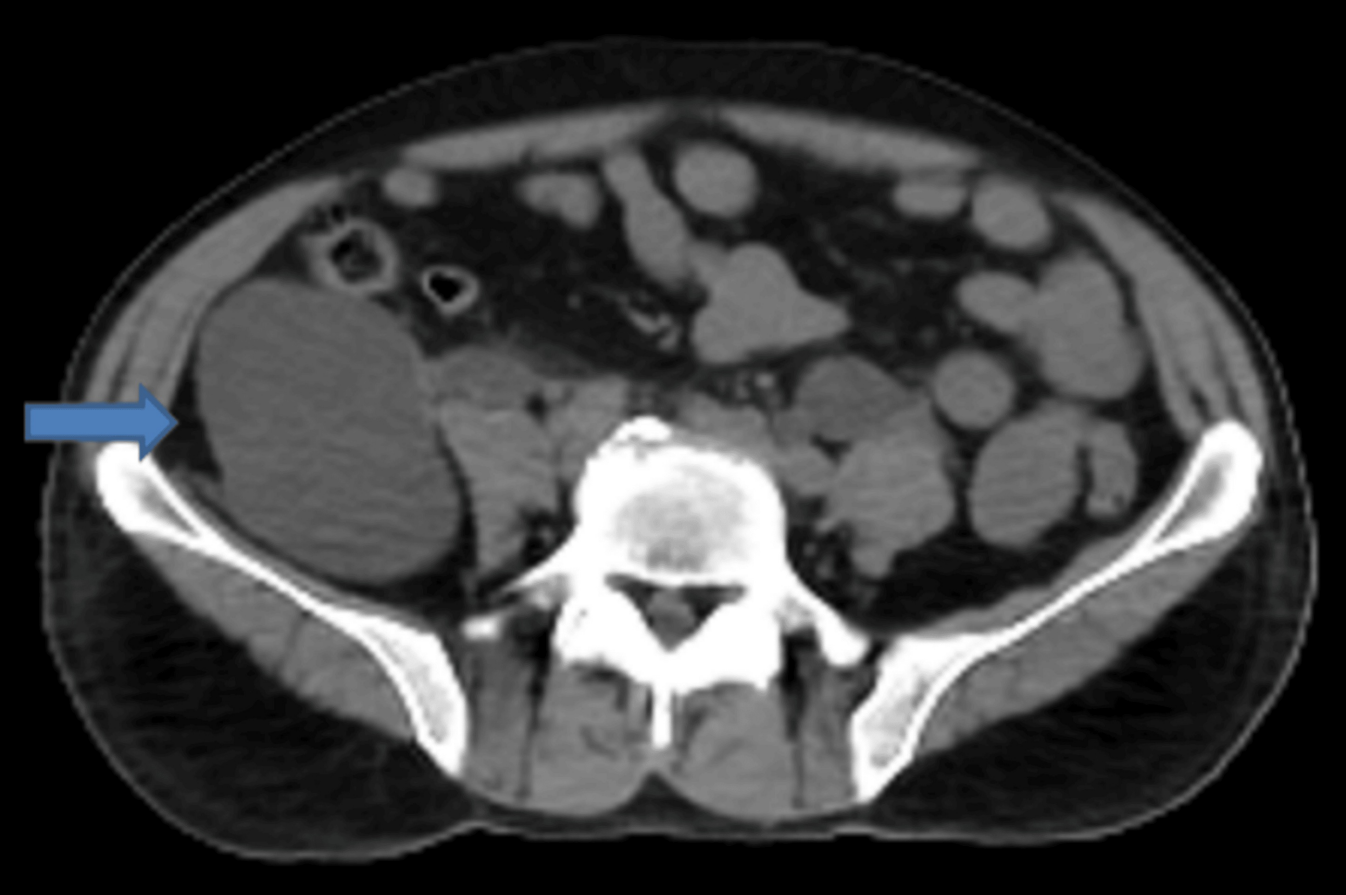
Non-contrast CT of the abdomen (axial view) showing an encapsulated fluid collection in the right lumbar region (blue arrow). The collection appears to be inseparable from the right ureter with moderate dilatation of the right collecting system.

**Figure 2:**
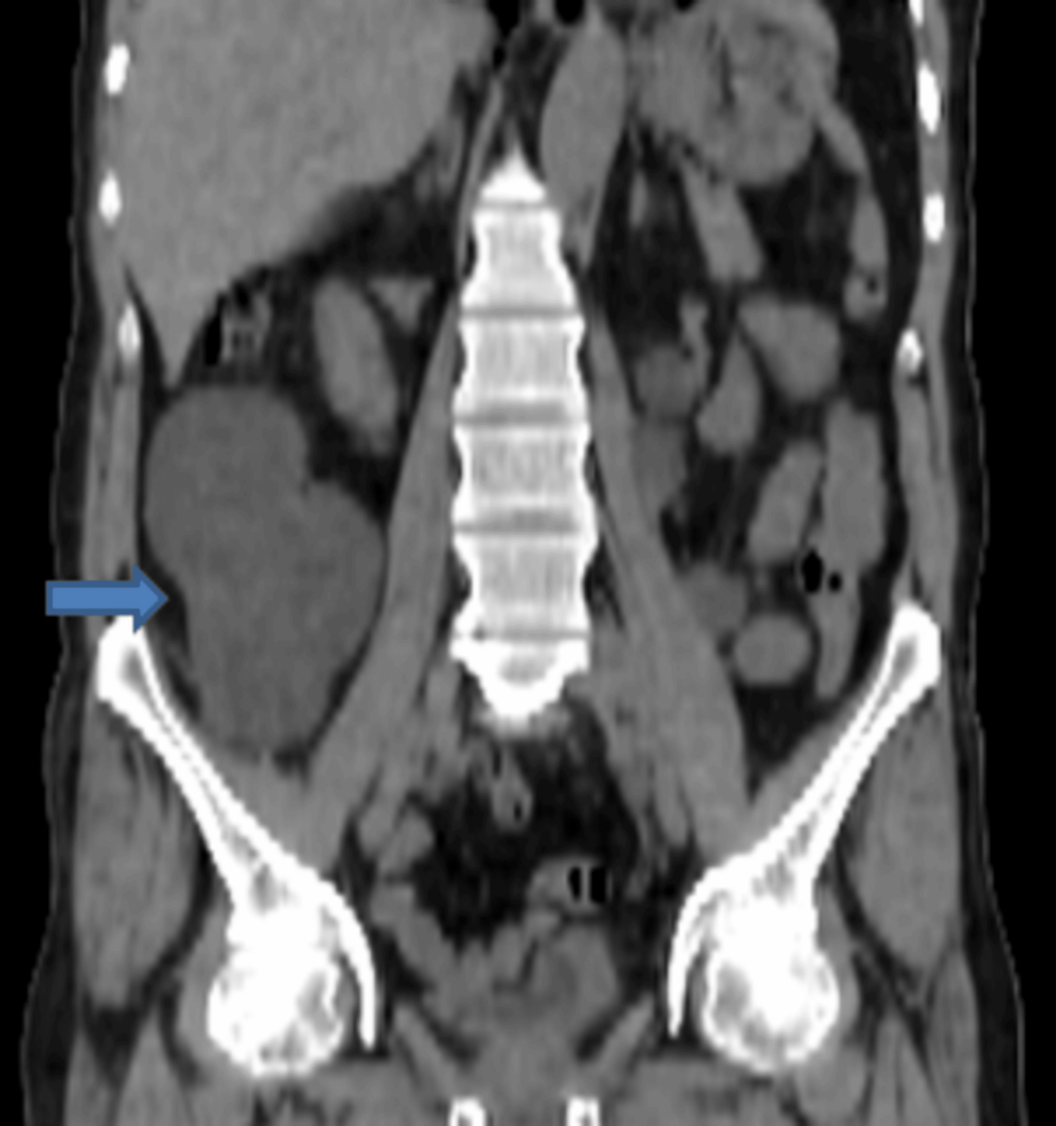
Non-contrast CT of the abdomen (coronal view) showing an encapsulated fluid collection in the right lumbar region (blue arrow). The collection appears to be inseparable from the right ureter with moderate dilatation of the right collecting system.

**Figure 3:**
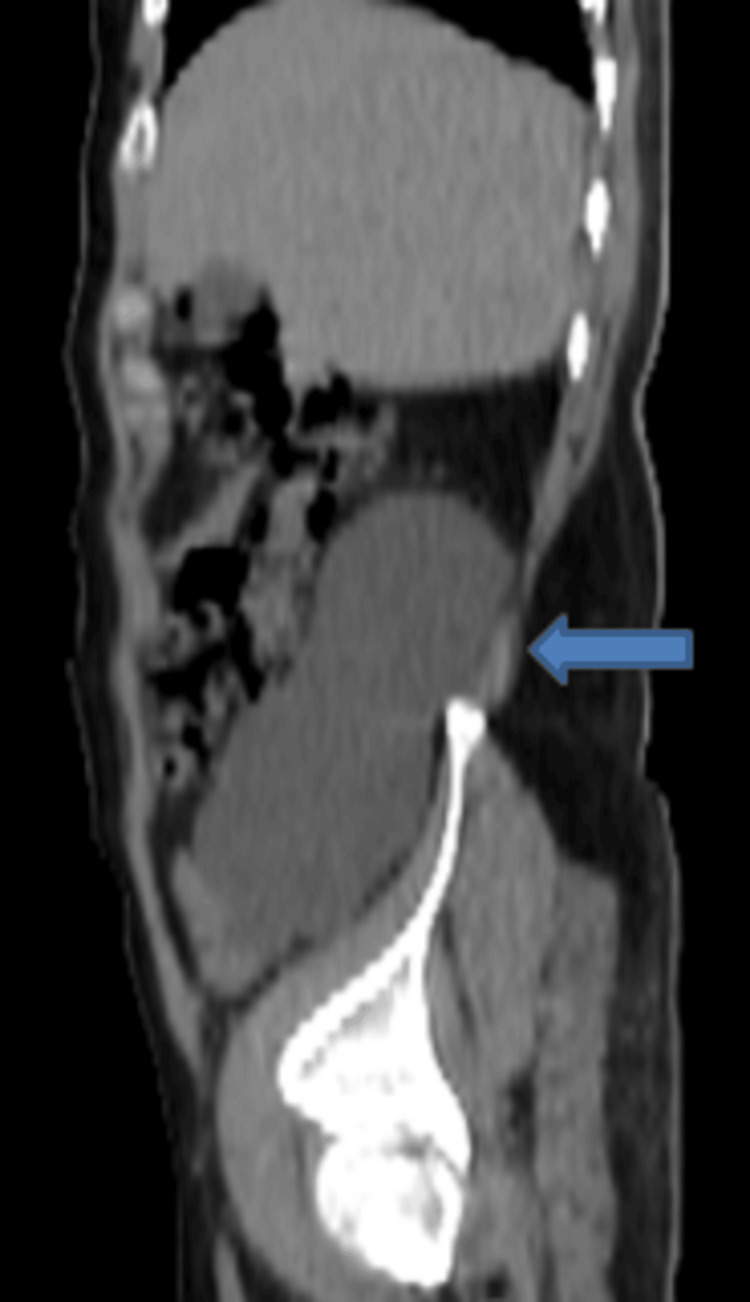
Non-contrast CT of the abdomen (sagittal view) showing an encapsulated fluid collection in the right lumbar region (blue arrow). The collection appears to be inseparable from the right ureter with moderate dilatation of the right collecting system.

An ultrasound was performed that revealed a large thin-walled area of anechoic fluid collection in the right lumbar region, apparently having no connection with the right kidney or the urinary bladder. Non-contrast CT scan of the abdomen and pelvis revealed that the collection was inseparable from the visualized right ureter (Figures 1-3).

**Figure 4:**
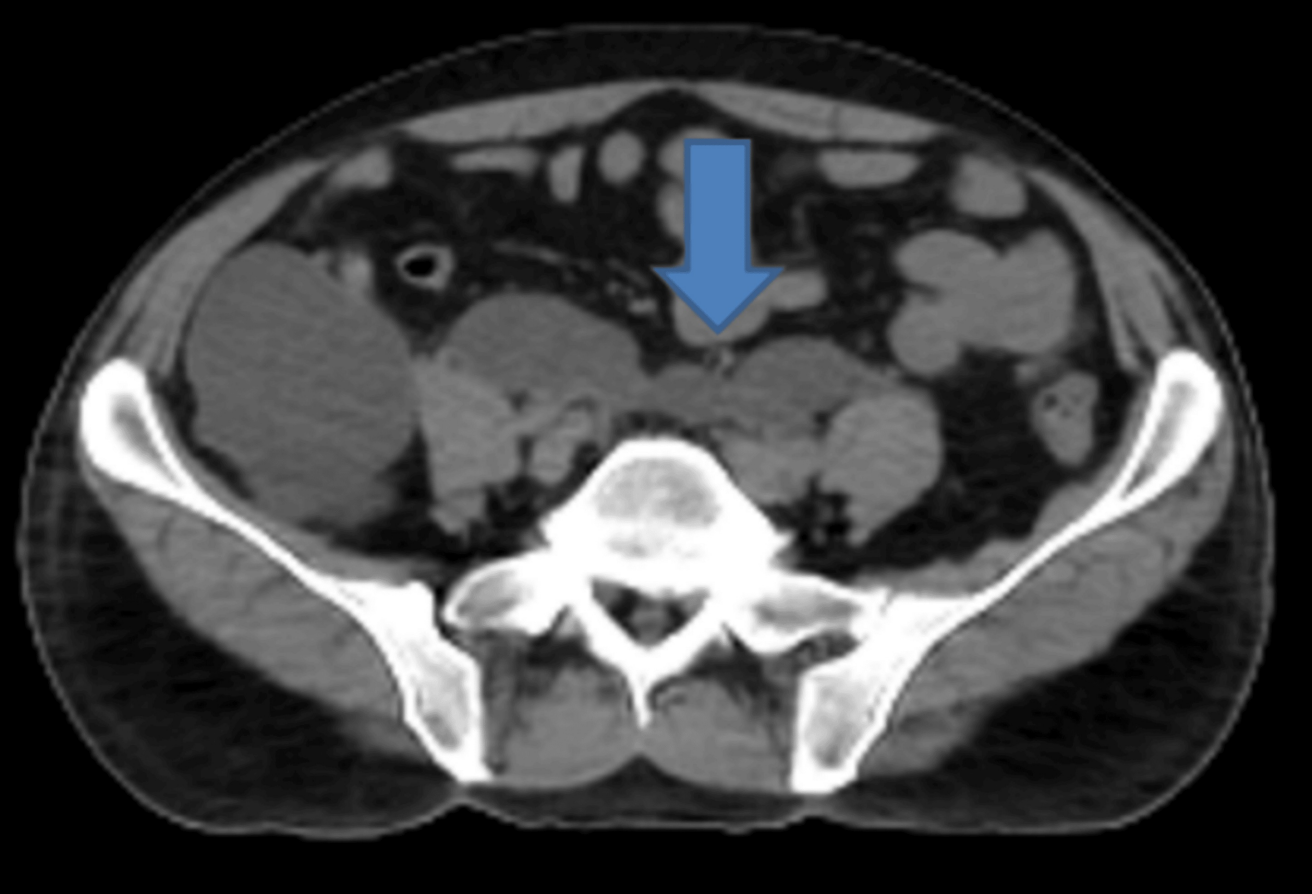
Non-contrast CT of the abdomen (axial view) showing an extension of the right lumbar region fluid collection across the midline (blue arrow) into the left lumbar region.

**Figure 5:**
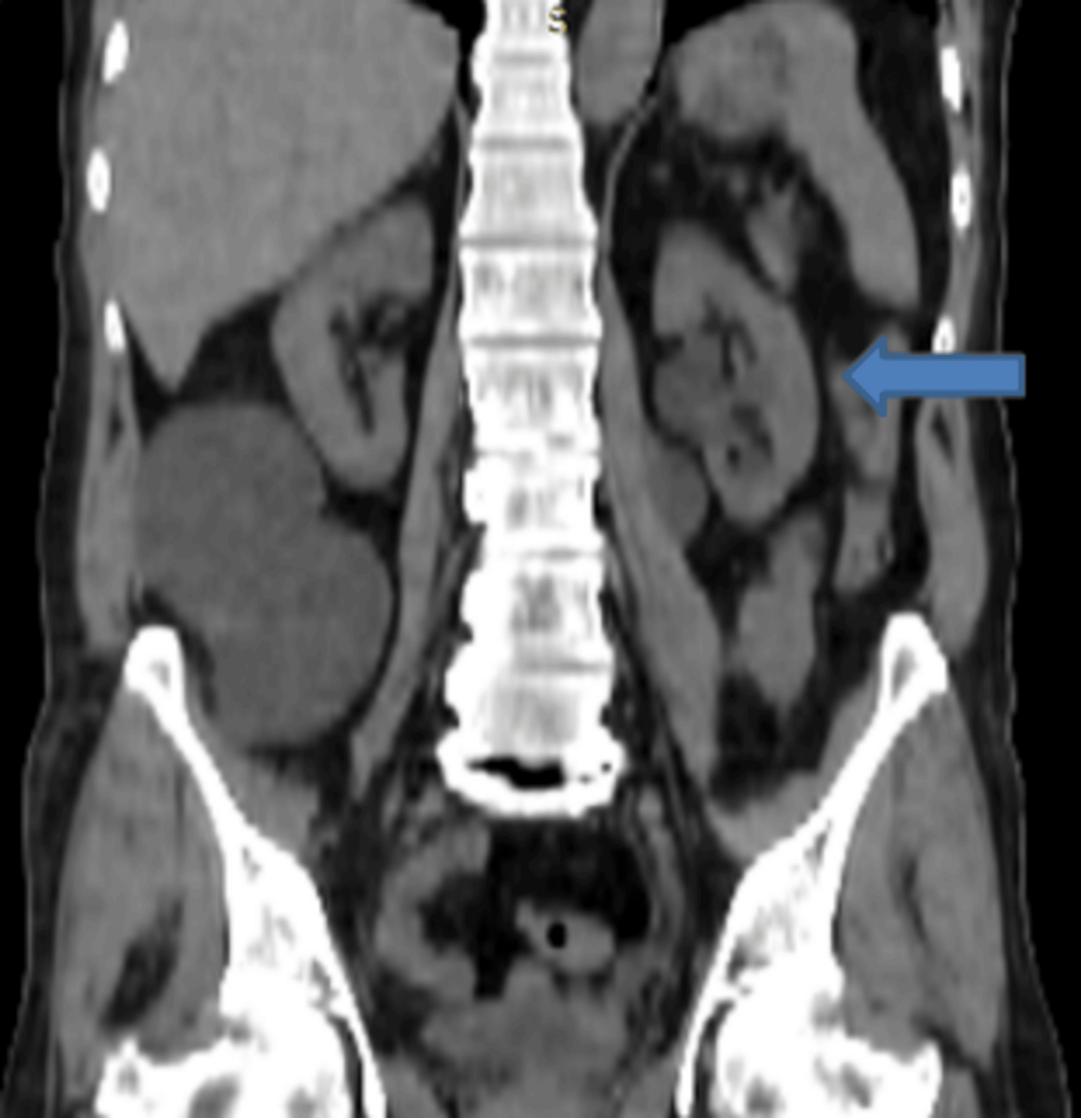
Non-contrast CT of the abdomen (coronal view) showing mild dilatation of the left renal collecting system (blue arrow).

This fluid collection also crossed the midline to extend into the left lumbar region (Figure 4). There was moderate dilatation of the right and mild dilatation of the left renal collecting system as well (Figure 5).

**Figure 6:**
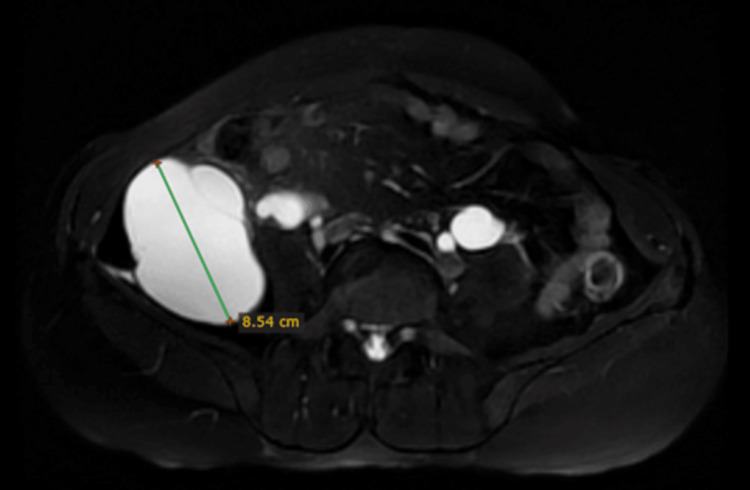
Non-contrast fat-saturated T2-weighted MRI of the abdomen (axial view) showing a hyperintense collection in the right lumbar region measuring 8.5 cm in the maximum anteroposterior dimension (blue arrow).

**Figure 7:**
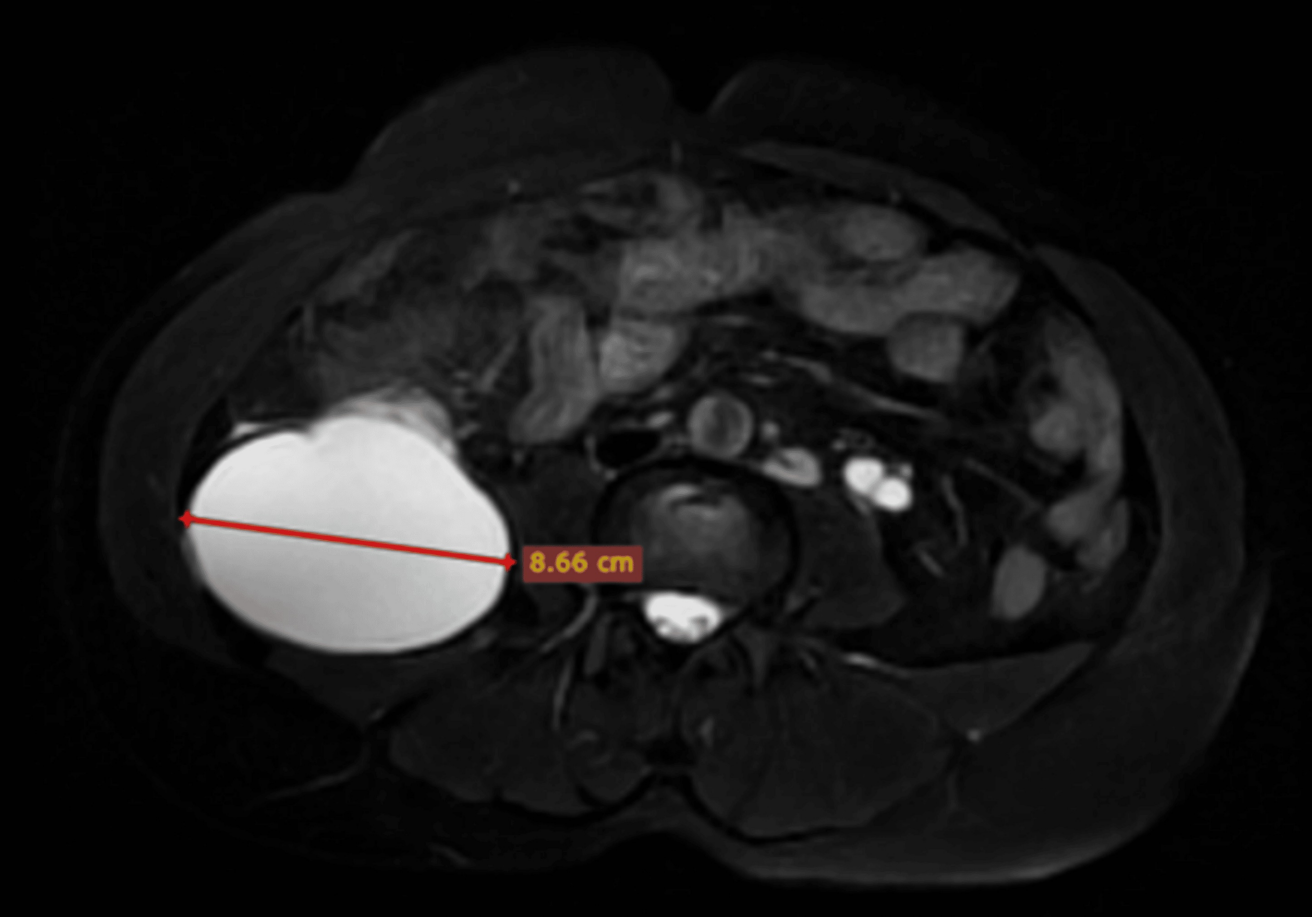
Non-contrast fat-saturated T2-weighted MRI of the abdomen (axial view) showing a hyperintense collection in the right lumbar region measuring 8.6 cm in the maximum transverse dimension (blue arrow).

**Figure 8:**
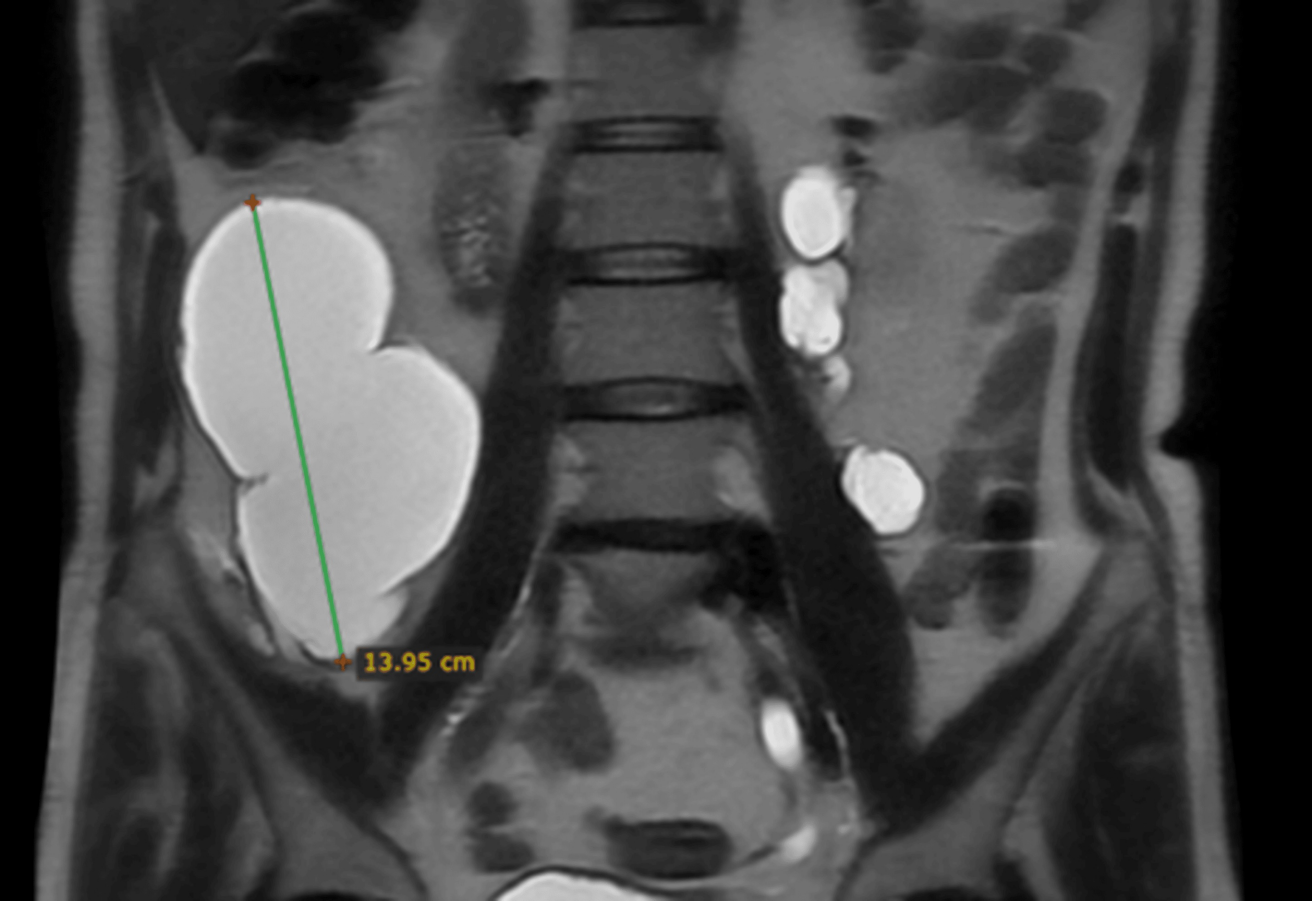
Non-contrast T2-weighted MRI of the abdomen (coronal view) showing a hyperintense collection in the right lumbar region measuring 13.9 cm in the maximum craniocaudal dimension (blue arrow).

To better delineate and localize the origin of this collection, an MRI was performed. An 8.5 × 8.6 × 13.9 cm (anteroposterior × transverse × craniocaudal) large encapsulated collection was seen returning hypointense signals on T1-weighted images and very hyperintense signals on T2-weighted images, suggesting simple fluid collection (Figures 6-8).

**Figure 9:**
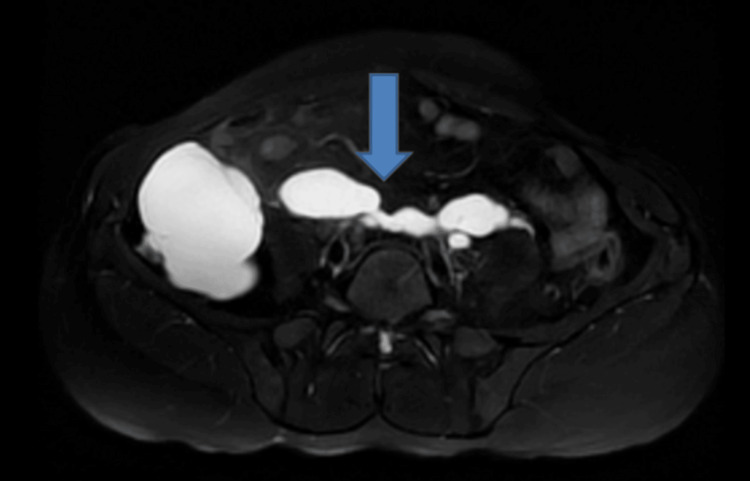
Non-contrast fat-saturated T2-weighted MRI of the abdomen (axial view) showing a hyperintense collection in the right lumbar region with extension across the midline into the left lumbar region (blue arrow).

**Figure 10:**
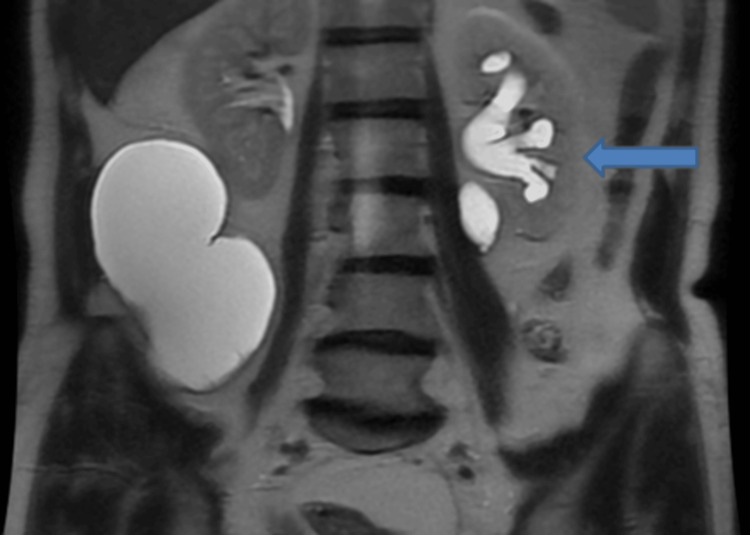
Non-contrast T2-weighted MRI of the abdomen (coronal view) showing dilatation of the left renal collecting system (blue arrow).

A small rent was observed in the right ureter which was the site of urinary leakage and the possible site of iatrogenic trauma during previous surgical interventions. Surrounding this breach was a large fluid collection that not only occupied the right lower abdomen and upper pelvic region but also crossed the midline to extend into the left lumbar region (Figure 9). Dilatation of the left renal collecting system was also seen (Figure 10).

**Figure 11:**
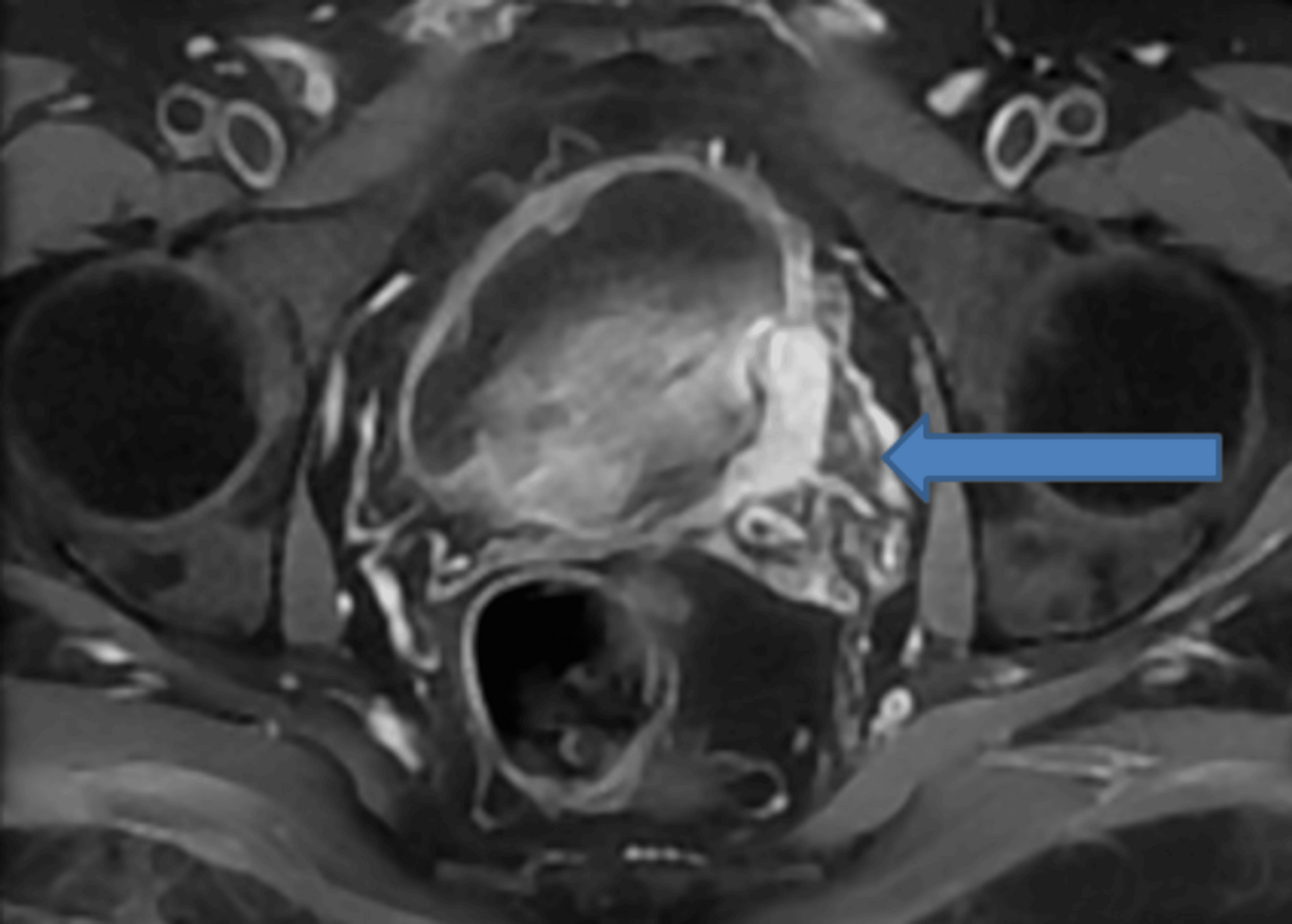
Contrast-enhanced T1-weighted MRI of the abdomen (axial view) showing enhancing soft tissue thickening along the left lateral urinary bladder wall (blue arrow) consistent with biopsy-proven transitional cell carcinoma.

Sonographic, CT, and MRI findings suggested the radiological diagnosis of urinoma. Irregular soft tissue growth was observed along the left lateral wall of the urinary bladder consistent with known transitional cell carcinoma (Figure 11). The patient was urgently referred to a urologist for further management and follow-up.

## Discussion

Urinoma is defined as an encapsulated collection of urine outside of the renal system, mostly in the peri-renal space and retroperitoneum. It can be spontaneous or traumatic. Increased intraluminal pressure within the renal collecting system secondary to renal stone, tumor, infection, or congenital abnormality results in spontaneous extravasation of urine [4]. Severe urinary tract obstruction can lead to a tear in the renal parenchyma, calyceal tear, or urinary bladder rupture that can lead to urinoma. Urinoma exhibits non-specific clinical symptoms in the form of diffuse or localized abdominal pain with or without fever, nausea, or vomiting. Physical examination findings of abdominal tenderness and signs of peritoneal irritation overlap with the diagnosis of appendicitis, ruptured ovarian cyst, diverticulitis, and cholecystitis [5].

Sonographically, urinoma appears as an anechoic cystic collection in the peri-renal space. Large urinoma can also have a mass effect on the adjacent kidney causing its displacement. Sometimes, decompression of the urinary tract is also seen post-rupture in the form of renal dysplasia or thickened trabeculated urinary bladder wall [6].

In utero, urinoma is associated with renal tract anomalies that lead to urinary tract obstruction, for example, pelvic-ureteric junction (PUJ) obstruction or posterior urethral valve. Urinoma associated with the posterior urethral valve demonstrates 75% preserved renal function compared with only 7% observed with PUJ obstruction, suggesting a worse prognosis in upper urinary tract obstruction [7].

Chan et al. reported a rare case of abdominal distension and respiratory distress in a newborn secondary to massive urinoma. A large retroperitoneal cystic collection was noted in the right peri-nephric space which on ultrasound-guided drainage yielded 500 mL of clear urine that subsequently led to symptomatic relief [8].

Possible differentials of a cystic peri-renal structure include mesenteric cyst, lymphangioma, enteric duplication cyst, hemorrhagic neuroblastoma, polycystic kidney disease, ureteric duplication, and cystic renal tumor. The integrity of adjacent intra-abdominal structures is very important to accurately determine the exact source of the fluid collection. One can easily confuse a small urinoma with a dilated calyx in the presence of hydronephrosis, but the presence of mass effect on renal parenchyma excludes dilated calyx. Thorough fluid evaluation helps in distinguishing urinoma from abdominopelvic ascites. Detailed patient history, clinical examination, and imaging are essential to confirm a diagnosis. Increased incidence of ureteral injuries has been observed in patients undergoing laparoscopic surgeries, especially for gynecological procedures. This can lead to the formation of urinoma in rare cases. Thus, a ureteral injury should be in the differentials of a patient presenting with acute abdomen post-laparoscopic surgery [9].

Renal trauma resulting in urinary extravasation is common. However, encapsulated urine collection in the form of urinoma is a rare event. Urinary leakage into the peri-renal space and retroperitoneum leads to the development of fibrous tissue forming peri-renal pseudocyst. Renal rupture can be renal parenchymal or of the renal collecting system. Any underlying anatomic abnormality makes kidneys susceptible to even minor trauma. Renal aneurysms and trauma are major causes of renal parenchymal rupture during pregnancy, with calculi being the most common cause of renal collecting system rupture. Chen et al. reported a rare case of spontaneous renal rupture resulting in peri-nephric urinoma in the third trimester of pregnancy [10].

Luu et al. described a case of secondary subcapsular urinoma in an adult male who was a known case of testicular embryonal carcinoma. One of the abdominal lymph nodes involved by the tumor resulted in ureteral involvement and ureteral rupture [5].

Diagnosis primarily relies on ultrasound. The presentation can be variable, most commonly including fluid areas in the peri-nephric space sometimes with floating ruptured kidney capsules, and discontinuous contour of the renal parenchyma or urinary tract. However, ultrasound holds some limitations regarding the detection and localization of small ruptures [11]. For this purpose, further imaging with intravenous urography, CT, or MRI is recommended. These complimentary imaging modalities can better estimate the size and location of urinoma and its relationship with surrounding structures. Misdiagnosis can delay the management and pose a threat to developing severe complications. Common potential outcomes of misdiagnosed urinoma include urinary peritonitis, hypertension, renal atrophy, and renal failure. Hence, this condition requires careful and serial monitoring and evaluation. Naito et al. reported a very rare case of spontaneous ureteral rupture and huge urinoma formation secondary to obstruction of the ureter by prostate cancer [12].

In our case, the second TURBT resulted in the removal of the tumor from the area adjacent to the right ureteropelvic junction possibly resulting in iatrogenic right ureteric injury with accumulation of extraureteral urine over time. It can also be secondary to recurrence of urothelial carcinoma.

Urinomas may be initially clinically occult but can lead to electrolyte disturbances and abscess formation if not properly diagnosed and managed [13]. Gültekin et al. incidentally discovered a case of iatrogenic urinoma on 18F-fluorodeoxyglucose positron emission tomography/computed tomography of a patient with urothelial carcinoma who had recently undergone endourological biopsy [14].

Treatment depends on the cause and is individualized for each case in an adequate clinical setting. Small urinomas require conservative management. However, in cases of large urinomas or non-resorbing urinomas, ultrasound or CT-guided drainage is performed as the risk of infection or sepsis is high in such cases. Hence, empiric antibiotic therapy should be considered initially to prevent the development of infected urinoma. In cases of continued urinary leakage despite image-guided drainage, insertion of a double-J tube or percutaneous nephrostomy tube is considered as it can provide quick drainage of leaked fluid and better relief of pressure symptoms [15].

## Conclusions

Iatrogenic ureteral injury leading to urinoma formation must be kept in the differential for patients presenting with acute abdominal pain, swelling, or fever after undergoing gynecologic, urologic, or retroperitoneal procedures. This potential differential must be considered by the physician when the patient complains of urinary retention despite urinary catheterization. Diagnosis of urinoma should not be delayed or missed as it carries significant morbidity. A high index of clinical suspicion, prompt imaging, and awareness of its characteristic features are crucial to avoid complications of abscess formation or hydronephrosis.
